# Anti-Obesity Effect of Chitosan Oligosaccharide Capsules (COSCs) in Obese Rats by Ameliorating Leptin Resistance and Adipogenesis

**DOI:** 10.3390/md16060198

**Published:** 2018-06-05

**Authors:** Haitao Pan, Chuhan Fu, Lanlan Huang, Yao Jiang, Xiaoyi Deng, Jiao Guo, Zhengquan Su

**Affiliations:** 1 School of Pharmaceutical Sciences, Sun Yat-Sen University, Guangzhou 510006, China; pangel7835001@163.com; 2 Guangdong Engineering Research Center of Natural Products and New Drugs, Guangdong Provincial University Engineering Technology Research Center of Natural Products and Drugs, Guangdong Pharmaceutical University, Guangzhou 510006, China; chuhanfu@163.com (C.F.); jiangyaoabcd@163.com (Y.J.); 1500718043DXY@gmail.com(X.D.); 3 Guangdong Metabolic Diseases Research Center of Integrated Chinese and Western Medicine, Guangdong Pharmaceutical University, Guangzhou 510006, China; 4 Guangdong Food and Drug Vocational Technical School, Guangzhou 510663, China; lanlanhuangle@163.com

**Keywords:** chitosan oligosaccharide, obesity, leptin, JAK2-STAT3, adipogenesis

## Abstract

Obesity is a global disease that causes many metabolic disorders. However, effective agents for the prevention or treatment of obesity remain limited. This study investigated the anti-obesity effect and mechanism of chitosan oligosaccharide capsules (COSCs) on rats suffering from obesity induced by a high-fat diet (HFD). After the eight-week administration of COSCs on obese rats, the body weight gain, fat/body ratio, and related biochemical indices were measured. The hepatic expressions of the leptin signal pathway (JAK2-STAT3) and gene expressions of adipogenesis-related targets were also determined. Our data showed that COSCs can regulate body weight gain, lipids, serum alanine aminotransferase, and aspartate aminotransferase, as well as upregulate the hepatic leptin receptor-b (LepRb) and the phosphorylation of JAK2 and STAT3. Meanwhile, marked increased expressions of liver sterol regulatory element-binding protein-1c, fatty acid synthase, acetyl-CoA carboxylase, 3-hydroxy-3-methylglutaryl-CoA reductase, adiponectin, adipose peroxisome proliferator-activated receptor γ, CCAAT-enhancer binding protein α, adipose differentiation-related protein, and SREBP-1c were observed. The results suggested that COSCs activate the JAK2-STAT3 signaling pathway to alleviate leptin resistance and suppress adipogenesis to reduce lipid accumulation. Thus, they can potentially be used for obesity treatment.

## 1. Introduction

Obesity is a chronically trophic metabolic disease mainly caused by an energy imbalance that leads to excess body fat accumulation. It has caused increasing concern in recent years. Obesity contributes to type 2 diabetes (T2D), hyperlipidemia, hypertension, cerebrovascular incidents, and cancers [1,2]. Recently, several anti-obesity drugs approved by the Food and Drug Administration (FDA) have been withdrawn from the market because of their unexpected adverse effects [3,4]. Currently, Orlistat is the only drug used as an over-the-counter treatment in weight loss aid worldwide, but its adverse effects, including gastrointestinal trauma and greasy feces, should not be ignored [4,5]. Therefore, it is necessary to develop natural products as alternative sources for weight loss agents due to their significant anti-obesity activity, novel structure, and potentially less severe side effects.

Chitosan oligosaccharide (M_N_ ≤ 1000 Da, COST) is a small molecular derivative of chitosan (CTS) with 2–10 polymerization degrees [6,7,8]. Besides non-toxicity to the human body, the absorption rate of COST in the intestinal tract is close to 100% due to its better water-solubility compared to that of CTS, which is slightly soluble in water [9,10,11]. The biological activities of COST are various, including anti-cancer, anti-inflammatory, hypoglycemic, anti-bacterial, and liver protective effects [11,12,13,14]. Additionally, a variety of studies have indicated that COST also exerts effective lipid-lowering and anti-obesity effects on obese animals, which makes it an effective lipid-lowering dietary supplement used in the field of food and nutrition to reduce lipid levels [10,14,15,16].

Leptin, a 16 kDa polypeptide that is primarily released from white adipose tissue (WAT), is an important hormone for weight management via the homeostatic control of energy equilibrium, thus facilitating the prevention of obesity [17,18]. Leptin exhibits its regulatory effect mainly by binding to the long-form leptin receptor-b (LepRb), which is widely distributed in the bodies of both humans and rodents [19,20]. JAK2-STAT3 (Janus kinase-2-signal transducer and activators of transcription-3) is the most important signaling pathway that mediates the energy balance regulation of leptin, which functions in many organs and tissues, such as the liver, brain, fat, muscle, and pancreas [21]. By binding to the extracellular domain of LepRb, leptin triggers the phosphorylation and activation of JAK2. The activated JAK2 recruits and phosphorylates STAT3 for its activation and translocation to the arcuate nucleus in the hypothalamus, which regulates the expression and neuronal excitability of POMC, AgRP, and NPY, ultimately suppressing appetite, promoting energy expenditure, and reducing body weight [19]. However, most obese patients show high levels of circulating leptin, which is attributed to leptin resistance that has occurred in the body [19]. In consequence, the drugs that improve leptin resistance will present significant anti-obesity effects.

In addition, Inoue et al. [22] revealed a deficiency of STAT3 in mouse liver aggravated steatosis and gluconeogenesis, whereas the overexpression of STAT3 reversed steatosis and suppressed the expression of the gluconeogenic gene. Meanwhile, Shi, et al. [23] showed that a hepatocyte-specific deficiency in JAK2 mice is required for the spontaneous development of steatohepatitis and glucose intolerance. Therefore, the activation of JAK2 or STAT3 in hepatocytes also ameliorates steatosis and improves lipid metabolism, which further contributes to anti-obesity action.

Adiponectin, an endogenous bioactive polypeptide secreted by adipocytes, is associated with the counteracting development of obesity and its related diseases. The gene expression and circulating levels of adiponectin are reported to be inversely correlated with obesity [24,25,26]. Sterol regulatory element-binding protein-1c (SREBP-1c) regulates de novo lipogenesis mainly in the liver via controlling the expression of its target gene, fatty acid synthase (FAS), a key enzyme for the formation of fatty acids [27,28]. With the increase of SREBP-1c expression, fatty acid metabolism becomes imbalanced, and subsequently the syntheses of hepatic triacylglycerol (TG) and total cholesterol (TC) are increased, which are closely associated with hepatic steatosis [29,30]. Acetyl-CoA is the precursor of fatty acid and cholesterol synthesis, and the reaction that translates acetyl-CoA into fatty acid or cholesterol is regulated by acetyl-CoA carboxylase (ACC), SREBPs, FAS, and so on [31]. In addition, 3-hydroxy-3-methylglutaryl-CoA reductase (HMGCR), the rate-limiting enzyme for hepatic cholesterol synthesis, catalyzes the conversion of HMG-CoA to mevalonate. A decreased expression of HMG-CoA is beneficial for suppressing the synthesis of hepatic cholesterol [32].

The hyperplasia and hypertrophy of adipocytes facilitate the development of obesity and its complications. Therefore, decreasing lipogenesis may benefit obesity prevention or treatment. Peroxisome proliferator-activated receptor γ (PPARγ) and CCAAT-enhancer binding protein α (C/EBPα) are two primary modulators of lipogenesis and regulate the proliferation, differentiation, and fat accumulation of adipocytes in WAT [33,34]. Adipose differentiation-related protein (ADRP), which covers the surface of phospholipid protein lipid droplets and is expressed early during the differentiation of adipose, stimulates fatty acid production and accelerates lipid droplet formation for fatty acid storage [34,35]. Meanwhile, FAS, SREBP-1c, and adiponectin also regulate lipid metabolism in WAT [36].

In this study, COST capsules (COSCs) were first prepared in capsule form due to several advantages, including the enhanced stability of COST manifested through strong hygroscopicity and oxidability, and the accelerated action rate of COST via the rapid dissolution, dispersion, and assimilation of the capsule in the gastrointestinal tract. Also, the capsule form can be easily identified and taken by most people. Subsequently, the anti-obesity effects of COSCs in a rat model exhibiting obesity induced by a high-fat diet (HFD) were investigated. Significantly ameliorated effects of COSCs on the development of obesity and its related complications were observed. To explore the anti-obesity mechanisms, the liver LepRb, JAK2, and STAT3 expressions in the leptin metabolic JAK2-STAT3 signaling pathway were evaluated by Western blotting, which suggested an improved leptin-resistant state via activating the JAK2-STAT3 signaling pathway. Moreover, the lipogenesis-related genes in the liver (including SREBP-1c, FAS, ACC, HMGCR, and adiponectin) and in adipose tissues (including PPARγ, C/EBPα, ADRP, FAS, and SREBP-1c) were also determined using quantitative RT-PCR analysis, indicating the adipogenesis suppression action of COSCs. These studies suggested that COSCs may be a prospective agent for obesity prevention or treatment.

## 2. Results

### 2.1. Food Intake, Body Weight, and Body Weight Gain

**Figure 1:**
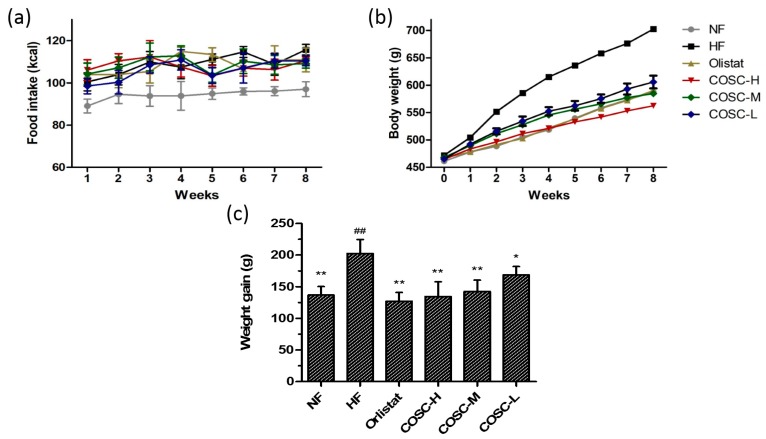
Chitosan oligosaccharide capsules (COSCs) reduced body weight gain of obese rats with no influence on food intake. Changes in the food intake (**a**); body weight (**b**), and body weight gain (**c**) during the eight-week treatment are shown. The data are expressed as means ± SE (*n* = 10). Note: * *p* < 0.05, ** *p* < 0.01 when compared to the high-fat (HF) group; ^##^ *p* < 0.01 when compared to the HF group.

To evaluate the anti-obesity effects of COSCs, food intake, body weight, and the body weight gain of the rats were measured over an eight-week period, the results of which are shown in Figure 1. For food intake, the high-fat (HF) group was slightly higher than the normal diet group (NF) and treatment groups during the experimental period, but the difference was not significant (Figure 1a), suggesting no influence of COSCs on the appetite of the rats, and the minor decrease of food intake in the treatment groups was attributed to a slight anorectic effect induced by HFD during the long-term HFD intake [10].

For the body weight gain, the average body weight of the HF group increased by 41.22% after eight weeks of feeding with HFD. Meanwhile, the eight-week administration of Orlistat, high (COSC-H), middle (COSC-M), and low dose of COS (COSC-L) significantly decreased body weight gain by 37.21%, 33.52%, 29.62%, and 16.61%, respectively (Figure 1b,c). Additionally, the effect of COSC-H on reducing weight gain was similar to that of the NF (32.47%) and Orlistat groups, suggesting effective weight loss action in the HFD-induced obese rats.

### 2.2. Serum Lipids and Leptin

**Figure 2:**
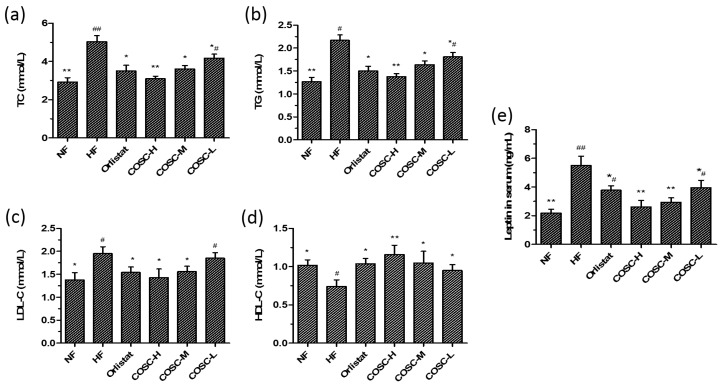
COSCs corrected serum lipid and leptin levels of high-fat diet (HFD)-induced obese rats. Serum TC (**a**); TG (**b**); LDL-C (**c**); HDL-C (**d**); and leptin (**e**) levels after eight weeks of treatment are shown. The data are expressed as means ± SE (*n* = 10). Note: * *p* < 0.05, ** *p* < 0.01 when compared to the HF group; ^#^ *p* < 0.05, ^##^ *p* < 0.01 when compared to the HF group.

Rat serum lipid levels after the eight-week administration were determined and are displayed in Figure 2. Compared with the NF group, 42.06%, 41.38%, and 29.23% increases of serum total cholesterol (TC), triglyceride (TG), and low-density lipoprotein cholesterol (LDL-C) levels, as well as a 25.20% decrease of serum high-density lipoprotein cholesterol (HDL-C), were shown in the HF group. Orlistat, COSC-H, COSC-M, and COSC-L could markedly lower serum TC by 30.36%, 38.49%, 28.37%, and 17.06% (Figure 2a), as well as TG by 30.88%, 36.41%, 24.42%, and 16.59% (Figure 2b), respectively. Moreover, COSCs, especially COSC-H and COSC-M, significantly ameliorated the increased LDL-C (Figure 2c) and reduced the HDL-C levels (Figure 2d) that were induced by HFD in the obese rats.

Various studies have indicated that leptin resistance, manifested by high levels of circulating leptin, is an important characteristic of nutritional obese patients [19]. In the study, the serum leptin of the HF group markedly increased by 60.36% when compared with that of NF group, suggesting that a leptin resistance state was induced by HFD in obese rats. Chitosan oligosaccharide capsule treatment significantly decreased the serum leptin level by 28.01~52.55% when compared to that of the HF group (Figure 2e), and this action was superior to that of Orlistat. All data in Figure 2 indicate that COSCs significantly improve serum lipids and leptin levels, facilitating lipids metabolism and ameliorating leptin resistance, which contribute to the anti-obesity activity of COSCs.

### 2.3. COSCs Facilitate Hepatoprotective Effects

**Figure 3:**
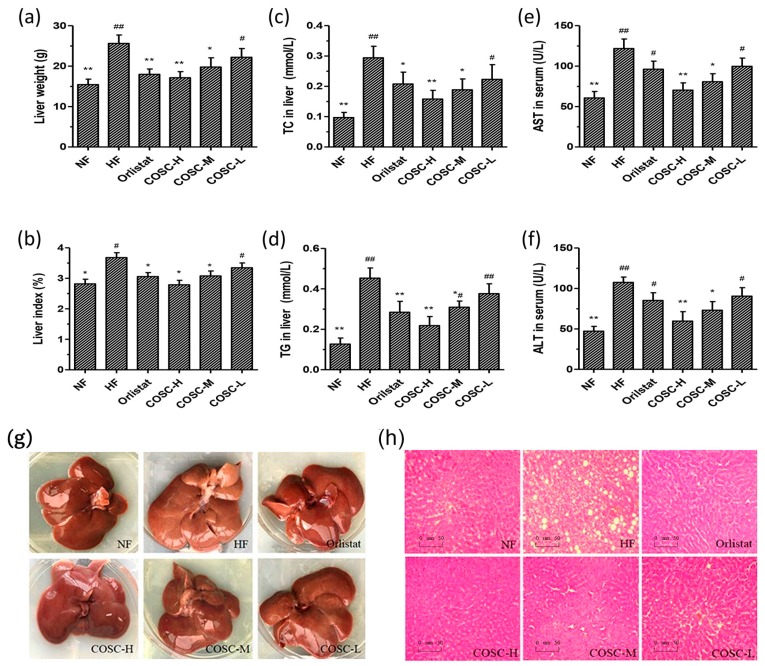
COSCs regulated hepatic lipids and facilitate hepatoprotective effects for obese rats. Liver weight (**a**); liver index (**b**); liver TC (**c**) and TG (**d**); as well as serum AST (**e**) and ALT (**f**) are shown; The whole liver (**g**); and liver histopathological slices (**h**) (200×) in different groups are also presented. Tissue sections were stained with hematoxylin and eosin (H&E). The data are expressed as means ± SE (*n* = 10). Note: compared with rats in the HF group, * *p* < 0.05, ** *p* < 0.01; compared with rats in the NF group, ^#^ *p* < 0.05, ^##^ *p* < 0.01.

The liver morphology, lipids, AST, and ALT levels are shown in Figure 3. Compared with the NF group, the average liver weight of rats fed HFD increased by 66.06%, which could be decreased by 13.55~33.12% after the administration of COSCs (Figure 3a). The corresponding results were also shown on the liver index (liver mass/body mass); that of the HF group was 30.50% higher than that of the NF group, although it could be decreased by 13.55~33.12% after the administration of COSCs (Figure 3b). For the liver TC (Figure 3c) and TG (Figure 3d) levels, the significant increases by 67.07% and 71.98% in the HF group were respectively shown when compared with that in the NF group, suggesting an obvious liver lipid accumulation induced by HFD. However, after the administration of Orlistat and the different doses of COSCs, the HFD-induced high levels of TC in the liver were significantly decreased by 29.35% (Orlistat), 46.34% (COSC-H), 35.93% (COSC-M), and 24.13% (COSC-L), and the levels of TG in the liver were also markedly decreased by 37.22% (Orlistat), 51.87% (COSC-H), 31.77% (COSC-M), and 17.63% (COSC-L).

Meanwhile, the liver morphology (Figure 3g) showed that the whole livers in the NF group were supple, bright red in color, smooth in the tunica of tissues, and characterized by sharp edges and a small volume. However, the livers of the HF group were slightly soft, dull pale in color, characterized by hypertrophic edges, and intumescent and distributed with white fat granules on the surface of the liver, suggesting that the severe fatty-liver-like illnesses had developed. The livers of the animals administered with the different doses of COSCs showed ameliorated hepatic steatosis, a color between bright red and dull pale, and reduced white fat granules. Histopathological slices of rat livers in the different groups were also observed after staining with hematoxylin and eosin (H&E) (Figure 3h). The liver slices revealed that there were no histological abnormalities of hepatocytes in the NF group with fewer fat droplets, whereas the hepatocytes in the HF group possessed serious fat vacuoles, indicating that the rats had developed a high degree of hepatic steatosis induced by HFD. Chitosan oligosaccharide capsules, especially COSC-H, can markedly decrease the fat vacuoles of hepatocytes whose cells are in alignment to varying degrees, similar to those of the NF group.

Liver injury, or hepatotoxicity, is the main relative factor of hyperlipidemia and obesity [37]. To check whether a hepatoprotective effect of COSCs presented, we measured the serum AST and ALT levels (Figure 3e,f). Obviously increased serum AST and ALT were shown in the HF group when compared to that in the NF group. Different doses of COSCs significantly lowered the activities of serum ALT and AST to the normal range. In particular, the effects of COSC-H and COSC-M were superior to those of Orlistat (*p* < 0.05), suggesting that COSCs play an important role in hepatoprotective function.

All data demonstrated that COSCs exert a hepatoprotective effect via decreasing the liver index, enhancing liver function, and relieving fatty liver, which contribute to the anti-obesity effects.

### 2.4. Fat Pad and Fat/Body Ratio

**Figure 4:**
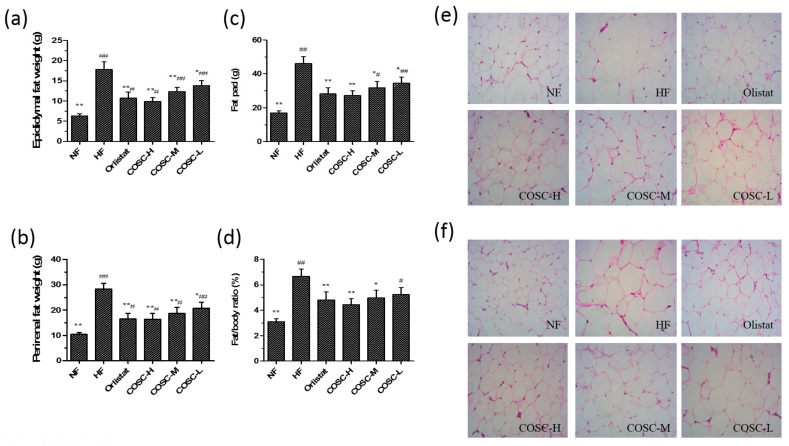
COSCs reduced the fat pad, fat/body ratio, and inhibit the accumulation of adipocytes in white adipose tissue (WAT) of obese rats. The epididymal (**a**) and perirenal (**b**) fat weight, fat pad weight (**c**), and fat/body ratio (**d**) are shown in the figure. The epididymal (**e**) and perirenal adipose tissue (**f**) slices (200×) are also shown. The numbers of epididymal or perirenal adipocytes were calculated in the same field of view. Tissue sections were stained with hematoxylin and eosin (H&E). Note: compared with rats in the HF group, * *p* < 0.05, ** *p* < 0.01; compared with rats in the NF group, ^#^ *p* < 0.05, ^##^ *p* < 0.01.

After the eight-week administration, the epididymal and perirenal WAT weights were measured to calculate the wet weight of the fat pad and fat/body ratio (Figure 4). Compared to the NF rats, the average weights of epididymal (Figure 4a) and perirenal WAT (Figure 4b) in the HF group were markedly increased by 64.38% and 63.19%. Also, COSCs were found to decrease the fat pad (Figure 4c, *p* < 0.05) and fat/body ratio (Figure 4d, *p* < 0.05) of rats dose-dependently to the normal range. Moreover, the inhibiting effects of COSC-H on HFD-induced elevated fat pad and fat/body ratio were slightly superior to that of Orlistat.

In addition, the histopathological results of epididymal (Figure 4e) and perirenal WAT (Figure 4f) showed that larger and distinctly hypertrophic adipocytes of rats in the HF group (70 epididymal adipocytes and 115 perirenal adipocytes under the 200× visual field) were observed when compared with those in the NF group (26 epididymal adipocytes and 29 perirenal adipocytes under the same field of view). Treatment with Orlistat and the three doses of COSCs inhibited the proliferation and size of adipocytes, as fewer and smaller adipocytes were observed compared to those in the HF group, using the same magnification and field of view. Therefore, COSCs can effectively exert anti-obesity effects by suppressing the growth and accumulation of adipocytes in WAT.

### 2.5. COSCs Activate Leptin Signaling Transduction

**Figure 5:**
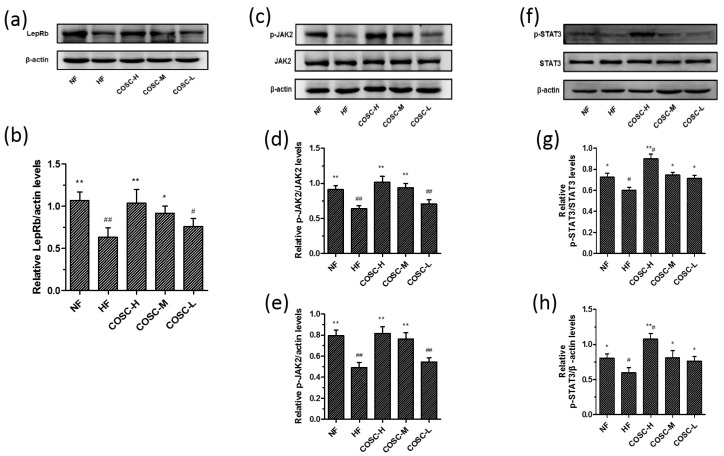
COSCs activated the JAK2-STAT3 signaling transduction of obese rats. Protein expression and quantification of LepRb (**a**,**b**); phosphorylation of JAK2 (p-JAK2, **c**–**e**) and phosphorylation of STAT3 (p-STAT3, **f**–**h**) in liver are shown here. The data are expressed as means ± SE (*n* = 10). Note: compared with rats in the HF group, * *p* < 0.05, ** *p* < 0.01; compared with rats in the NF group, ^#^ *p* < 0.05, ^##^ *p* < 0.01.

Leptin resistance, characterized by elevated circulating leptin levels and decreased leptin sensitivity, is the central mechanism for the development of obesity. Attenuation of the JAK2-STAT3 signaling transduction pathway can be considered as the crucial risk factor for leptin resistance. Therefore, the improvement of leptin resistance by regulating the JAK2-STAT3 signaling pathway effectively exerts anti-obesity activity [19,38]. In this study, the protein levels of liver LepRb, JAK2, p-JAK2, STAT3, and p-STAT3 in the activation of the JAK2-STAT3 signal pathway were detected using Western blotting, as displayed in Figure 5. The eight-week treatment of COSC-H and COSC-M significantly reversed the lower expression of LepRb in HFD-induced obese rats (Figure 5a,b). The reduced phosphorylation of JAK2 (Figure 5c–e) and STAT3 levels (Figure 5f–h) in the HFD group were also prominently upregulated by COSCs (especially COSC-H and COSC-M), indicating the improvement of the leptin resistance state. Therefore, COSCs can ameliorate leptin resistance via upregulating LepRb and activating the JAK2-STAT3 signaling pathway, contributing to the improvement of obesity.

### 2.6. COSCs Regulate the Expression of Lipogenesis-Related Genes

**Figure 6:**
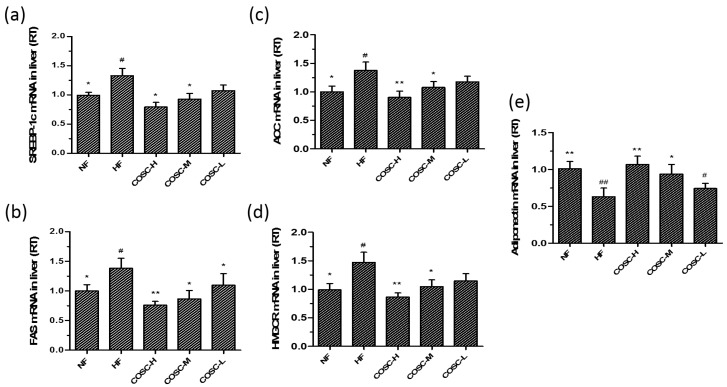
COSCs regulated the expression of liver lipogenesis-related genes in obese rats. The liver mRNA levels of *SREBP-1c* (**a**); *FAS* (**b**); *ACC* (**c**); *HMGCR* (**d**); and *adiponectin* (**e**) were detected by Q-PCR. ∆Ct is the average value of 10 samples in the formulation (average mRNA expression of experiment groups/average mRNA expression of the NF group) = 2^−∆∆Ct^ = 2^(−∆Ct control −∆Ct FF)^. If 2^−∆∆Ct^ < 1, the average mRNA expression of the experiment groups is lower than that in the NF group. If this value is higher than 1, the average mRNA expression of the experiment groups is higher than that in the NF group. The data are expressed as means ± SE (*n* = 10). Note: compared with rats in the HF group, * *p* < 0.05, ** *p* < 0.01; compared with rats in the NF group, ^#^ *p* < 0.05, ^##^ *p* < 0.01.

The liver lipogenesis-related genes, *SREBP-1c*, *FAS*, *ACC*, and *HMGCR*, as well as the WAT lipogenesis-related genes, *adiponectin*, *PPARγ*, *C/EBPα*, *ADRP*, *FAS*, and *SREBP-1c*, were also measured (Figure 6a–e). Compared with the NF group, the significant upregulation of *SREBP-1c*, *FAS*, *ACC*, and *HMGCR* as well as the downregulation of *adiponectin* were observed in the HF group. Chitosan oligosaccharide capsules, especially the high and middle doses, reversed the upregulated expression of *PPARγ*, *C/EBPα*, *ADRP*, *FAS*, and *SREBP-1c*, and the downregulation of *adiponectin* induced by HFD in obese rats, indicating an improved hepatic lipid metabolism by inhibiting lipid synthesis in the liver.

**Figure 7:**
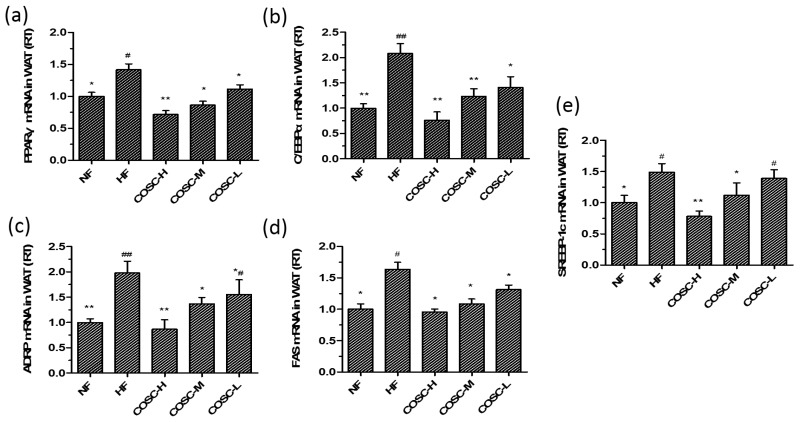
COSCs regulated the expression of lipogenesis-related genes in obese rat WAT. The mRNA levels of *PPARγ* (**a**); *C/EBPα* (**b**); *ADRP* (**c**); *FAS* (**d**); and *SREBP-1c* (**e**) in epididymal adipose tissues were quantified by Q-PCR. The 2^−∆∆Ct^ method was used for the quantification of each mRNA as shown in Figure 6. The data are expressed as means ± SE (*n* = 10). Note: compared with rats in the HF group, * *p* < 0.05, ** *p* < 0.01; compared with rats in the NF group, ^#^ *p* < 0.05, ^##^ *p* < 0.01.

White adipose tissue is an important tissue for ensuring energy storage and fat mobilization. However, the hyperplasia and hypertrophy of WAT will lead to metabolic syndromes, and the inhibition of adipogenesis can be an effective strategy for obesity treatment [15]. We measured lipogenesis-related genes, including *PPARγ*, *C/EBPα*, *ADRP*, *FAS*, and *SREBP-1c*, in the epididymal adipose tissues (Figure 7). The remarkable downregulation of *PPARγ*, *C/EBPα*, *ADRP*, *FAS*, and *SREBP-1c* were also observed to be correlated with the dose of COSCs.

All of these results suggest that COSCs can inhibit adipogenesis in liver and WAT by regulating the expressions of related genes involved in lipid synthetic metabolism to ameliorate obesity and its related metabolic diseases.

## 3. Discussion

Obesity-related metabolic disorders, including type 2 diabetes, hyperlipidemia, hypertension, cerebrovascular incidents, and cancers, are classified as endocrinology diseases by the World Health Organization (WHO) [1,39]. Obesity, paralleling the acquired immune deficiency syndrome (AIDS), drugs, and alcoholism, is regarded as one of four medical social issues worldwide that are becoming a ponderous burden to public health system [40]. Therefore, it is urgent to develop new types of anti-obesity drugs. Natural products are promising alternative sources due to their effective biological activities and potentially less severe side effects [14].

Several studies by others and our group have demonstrated that COST has anti-obesity effects and is a promising anti-obesity agent [15,16,41]. In the current study, we prepared COST in a capsule form and investigated its anti-obesity effects and molecular mechanisms.

The obese animal model used in the experiment showed obviously increased body weight gain and lipid accumulation as well as elevated organ index, as reported before the anti-obesity treatment [42,43]. The obese rats were treated with COSCs for eight weeks, and the obese evaluation indicators, mainly including body fat pad weight, fat/body ratio, liver and serum lipids, as well as liver and adipose tissue slices, were determined. In agreement with previous experiments [9,10], COSCs showed obvious inhibition of increases in rat body weight (Figure 1c), liver index (Figure 3a,b), body fat (Figure 4c), and the proportion of fat to body weight (Figure 4d) without influence on the appetite, as well as reducing adipocyte hypertrophy and fat accumulation (Figure 4e,f), suggesting significant anti-obesity effects. Simultaneously, COSCs can markedly reverse HFD-induced serum high levels of TC, TG, and LDL-C as well as low HDL-C levels in a dose-dependent manner, which also contribute to its anti-obesity actions (Figure 2).

Hepatic steatosis (fatty liver), induced by excessive lipid accumulation in the liver, is highly relevant to obesity and its related complications [44,45]. In this study, COSCs effectively lowered the high weight and index of the liver, and reversed HFD-induced intumescent, pale, and slightly soft livers as well as diminished fat vacuoles, indicating the ameliorating effect of COSCs on hepatic steatosis mainly achieved by reducing excessive fat accumulation (Figure 3). Meanwhile, the high levels of hepatic TC and TG were also reversed by COSCs, especially COSC-H and COSC-M, indicating that a decrease in excessive hepatic lipids improved liver lipid metabolism (Figure 3), similar to the previous study of COS, which promotes the reverse cholesterol transport (RCT) process for the excretion of liver lipids [10]. Together with the decreased serum AST and ALT of the treatment groups, COSCs exerted liver protective effects.

Leptin resistance, characterized by elevated circulating leptin levels and decreased leptin sensitivity, is the central mechanism for the development of obesity. Attenuation of the JAK2-STAT3 signaling transduction pathway is a crucial risk factor for leptin resistance [19,20]. Chitosan oligosaccharide capsules significantly reduced circulating leptin in obese rats, and improved the leptin resistance state by upregulating the expression of LepRb and the phosphorylation levels of JAK2 and STAT3 (Figures 2e and 5). The activation of the JAK2-STAT3 signaling pathway also contributes to the remissions of steatosis and dyslipidemia of obese rats [22,23]. Therefore, the improvement of leptin resistance by regulating the JAK2-STAT3 signaling pathway is the mechanism by which COSCs exerts effective anti-obesity activity in HFD-induced obese rats.

SREBP-1c, FAS, ACC, and HMGCR are the crucial factors that influence lipid metabolism in the liver. SREBP-1c increases the activity of the key enzyme FAS and regulates the synthesis of TC and TG when free fatty acids are released into hepatocytes [29]. Acetyl-CoA carboxylase catalyzes the conversion of acetyl-CoA to Malonyl-CoA, which regulates the rate of fatty acid synthesis in the first stage [31]. An increase of HMGCR expression led to cholesterol synthesis in the liver due to the fact that HMGCR is a rate-limiting enzyme for hepatic cholesterol synthesis and catalyzes HMG-CoA to be converted to mevalonate [32]. Additionally, adiponectin acts as the negative regulator factor of adipogenesis, counteracting the development of fatty liver and obesity [24,26]. Chitosan oligosaccharide capsules can inhibit hepatic lipid synthesis by prominently regulating the gene expressions of SREBP-1c, FAS, ACC, HMGCR, and adiponectin to improve hepatic steatosis and lipid metabolism disorder, thereby exerting hepatoprotective and anti-obesity effects (Figure 6).

In addition, the expressions of genes involved in WAT were also determined to evaluate the influences of COSCs on adipogenesis in WAT. PPARγ, together with C/EBPα, can promote the proliferation and differentiation of adipocytes and control lipid storage in WAT [33,46]; ADRP can accelerate the formation of fatty acids and lipid droplet accumulation in the early differentiation of adipocytes [34,47]; FAS and SREBP-1c facilitate the synthesis of TC and TG of WAT [48]. Unsurprisingly, the gene expressions of PPARγ, C/EBPα, ADRP, FAS, and SREBP-1c in the epididymal adipose tissues were upregulated after the administration of COSCs, which contribute to the ameliorated effect of hyperplasia or hypertrophy of adipocytes to suppress adipogenesis in WAT (Figure 7).

In summary, COSCs could be used as a drug candidate with a good ability to control obesity. Chitosan oligosaccharide capsules can activate the JAK2-STAT3 signaling pathway to alleviate leptin resistance and inhibit adipogenesis by regulating the relevant adipocytokines. Therefore, COSCs may serve as a natural product prospective agent for obesity prevention and treatment.

## 4. Materials and Methods

### 4.1. Materials and Supplies 

Chitosan oligosaccharide (M_N_ ≤ 1000 g/mol; deacetylation degree, 95.6%) was obtained from Laizhou Haili Biological Products Co. Ltd. Laizhou, Shandong, China. The COSCs used in this work were prepared in our laboratory. The Orlistat capsules were provided by Chongqing Fortune Pharmaceutical Co. Ltd., Chongqing, China. Total cholesterol, TG, HDL-C, LDL-C, AST, and ALT kits were provided by BioSino Biotechnology and Science Inc., Beijing, China. The BCA protein quantified assay kit (Cat. No. P0012) was provided by Beyotime Biotechnology Inc., Shanghai, China. The rabbit polyclonal anti-LepRb (Cat. No. 20966-1-AP), anti-JAK2 (Cat. No. 17670-1-AP), and anti-STAT3 (Cat. No. 10253-2-AP) antibodies were purchased from Proteintech, Inc., Wuhan, China. The rabbit monoclonal anti-JAK2 (Cat. No. ab219728) and anti-STAT3 (Cat. No. ab76315) phosphorylated antibodies were purchased from Abcam Co. Ltd., Cambridge, UK. The rabbit anti-β-actin (Cat. No. bs-0061R) and Goat anti-rabbit IgG/HRP (Cat. No. bs-0295G-HRP) antibodies were purchased from Biosynthesis Biotechnology Co., Ltd., Beijing, China. The reverse transcription kit (PrimeScript™ RT reagent kit with gDNA Eraser (Cat. No. RR047A)) and the PCR kit (SYBR Premix Ex Taq™ kit (Cat. No. RR420A)) were all supplied by TaKaRa Inc. (Otsu, Japan). All of the other reagents were of analytical grade and were used without further purification.

### 4.2. COSCs Preparation

The COSCs were prepared as shown in Reference [49]. Briefly, COST and 5% povidone (PVP) dissolved in anhydrous ethanol were ground equally in a mortar using the appropriate proportions. Then, the soft materials were made into granules using an oscillating granulator (20 mesh sieves) and desiccation. The dry granules were filled in a 1^#^ hollow capsule shell using a manual capsule filling plate, and the loading amount was 300 mg per capsule.

### 4.3. Animals and Diets

Ninety-five 5-week-old male specific pathogen free (SPF) Sprague–Dawley (SD) rats (weight, 120~150 g; age, 5 weeks) were provided by the Guangdong Medical Laboratory Animal Center (GMLAC, Guangzhou, China) and were maintained in an SPF room (temperature: 22~25 °C, related humidity: 50~60%, differential pressure: ≥10 Pa, under a constant day-night rhythm). Water was given to rats freely throughout the experiments. The welfare of the animals in the in vivo experiments was handled strictly following “Guidelines on kindly treatments for experimental animals” by the Science and Technology Ministry of China (2006) 398. All protocols were approved by the Institutional Animal Ethics Committee of Guangdong Pharmaceutical University (Approval No. gdpulac 2018092). All animals were fed with the normal diet (Guangdong Pharmaceutical University Laboratory Animal Center, Guangzhou, China) for one week. Subsequently, the 95 rats were divided into two groups: the normal diet group (NF) with 10 rats fed the normal diet, and the HFD group (HF), with 85 animals fed the HFD to obtain the obese model.

The HFD was comprised of 54% basic feed, 15% lard, 15% sucrose, 4% milk powder, 3% peanut, 5% egg yolk powder, 1% sesame oil, 2% salt, 0.6% dicalcium phosphate, and 0.4% mountain flour in SPF packaging (provided by GMLAC (No. 20150925)). After two weeks of feeding with HFD, two thirds of the rats were sorted by body weight gain. The excluded one third of the rats that gained less weight were kept on the HFD for another six weeks. The obese model was regarded as being accomplished when the average weight of the rats fed with HFD surpassed 20% of that of the basic fed rats.

The 50 obesity-sensitive rats were then randomly and equally divided into five groups as follows: (1) HFD group fed with HFD ad libitum only (HF); (2) HFD group treated with Orlistat (75 mg/kg·day) (Orlistat); (3–5) HFD group supplemented with low (150 mg/kg·day, COSC-L), middle (300 mg/kg·day, COSC-M), and high (600 mg/kg·day, COSC-H) dose COSCs. Because the capsules and COST were all water-soluble, the COST capsules were consequently dissolved in distilled water, which formed the COST solution with a constant concentration of 600 mg/mL. The corresponding samples to be tested should be solubilized in distilled water and administered by oral gavage daily with a dose of 1 mL/100 g (the rats’ body weight) at the same time for eight weeks until the experiment ended. The rats in the HF and NF groups were simultaneously administered equal amounts of distilled water in the same way as described above. All rats obtained food and water freely during the experimental period, and were sacrificed at the age of 22 weeks.

### 4.4. Experimental Design

#### 4.4.1. Determination of Food Intake and Weight Gain

The 24-h food intake levels of rats were recorded daily, while body weight, body length, and abdominal girth were determined every week during the animal study.

#### 4.4.2. Determination of Serum Lipid and Leptin Levels

The rats were fasted for 16 h and then subjected to 1% sodium pentobarbital (0.5 mL/100 g body weight) anesthesia, and their blood was collected from the aorta abdominals. The serum was obtained via centrifuging at 3500 rpm for 30 min at 4 °C, and the serum lipid and leptin levels were then measured. The concentrations of TC, TG, HDL-C, LDL-C, AST, and ALT were measured with commercial assay kits using an automated biochemistry analyzer BC200 instrument. The concentration of leptin were measured using an enzyme-linked immunosorbent assay (ELISA) kit (R&D, Systems China Co. Ltd., Shanghai, China), and the absorbance was detected at 450 nm using a multifunctional Berthold Mithras LB940 microplate reader (Berthold Technologies GmbH & Co. KG, Bad Wildbad, Germany).

#### 4.4.3. Determination of Fat Pad, Fat/Body Ratio, and Visceral Index

Rats were subjected to ether anesthesia, sacrificed, and necropsied after serum preparation. Then the liver, epididymal WAT, and perirenal WAT were quickly stripped and weighed by an electronic scale at 4 °C. After a picture was taken of the whole liver from each animal, the total wet weight of liver, epididymal fat, and perirenal fat were used to determine the liver index, fat pad, and fat/body ratio, respectively. The tissues were promptly stored at −80 °C for further analysis following their removal.

#### 4.4.4. Determination of Hepatic Lipids, AST, and ALT

A piece (approximately 0.1 g) of liver tissue was homogenized in 0.9 mL 0.9% sodium chloride, and the supernatant was collected after centrifuging at 2500 rpm for 10 min at 4 °C. The hepatic TC, TG, AST, and ALT levels were measured with commercial assay kits, as was performed for serum lipids previously.

### 4.5. Histology of the Different Tissues

The liver, white epididymal, and perirenal WAT were cut into 0.6 cm^3^, then were rinsed with normal saline and put in a tissue cassette. The cassettes were marked with a pencil and then placed into a 12% formaldehyde solution for 24 h to fix the tissue. After the residual fixative was cleaned with distilled water, the tissues were firstly dehydrated via 30%, 50%, 70%, 80%, 90%, 95%, and 100% ethanol, then embedded in paraffin (BMJ-III embedding machine, Changzhou Electronic Instrument Factory, Jiangsu, China) and lastly cut into 5-μm-thick sections using a Leica RM2235 microtome (Leica, Heidelberg, Germany). The tissues were stained with hematoxylin and eosin (H&E) and observed under a microscope at 200× magnification.

### 4.6. Western Blotting Assays

Total protein was isolated from the liver tissues (25 mg) with 0.50 mL of cold RIPA Lysis Buffer (50 mM Tris (pH 7.4), 150 mM NaCl, 1% Triton X-100, 1% sodium deoxycholate, 0.1% SDS, and protease and phosphatase inhibitor), followed by centrifugation twice at 12,000 *g* for 30 min at 4 °C. The protein concentrations were quantified by the Beyotime BCA protein assay kit. The isolated proteins were diluted to the same protein concentrations. An equal amount of each protein lysate (35 μg) was subjected to sodium dodecyl sulfate-polyacrylamide gel electrophoresis (SDS-PAGE) on 10% gels. After electrophoresis, the gels were then transferred to polyvinylidene fluoride membranes (Millipore, USA). After blocking with 5% bovine serum albumin (BSA) in TBST buffer (25 mM Tris, 150 mM NaCl, 0.05% Tween 20, pH 7.4) for two hours, the membranes were correspondingly incubated for 2 h with primary antibodies, including rabbit polyclonal anti-LepRb, anti-JAK2, and anti-STAT3 antibodies, and rabbit monoclonal anti-JAK2 and anti-STAT3 phosphorylated antibodies, as well as rabbit anti-β-actin antibody, in 5% skim milk. The secondary antibody (Goat anti-rabbit IgG linked HRP) was incubated with the membranes for another 1 h after washing with TBST buffer. The membranes were developed with Pierce’s West Pico chemiluminescence substrate (Millipore, Burlington, MA, USA) after washing with TBST buffer. The chemiluminescence imaging system (Sage Creation, Beijing, China) and its Lane 1D gel image software were applied to quantify the grayscale of the protein bands with the value of the β-actin band as an internal reference.

### 4.7. Reverse Transcription-Polymerase Chain Reaction (RT-PCR) Analysis

Total RNA was isolated from rat livers or white epididymal adipose tissues using the TRIzol reagent (Invitrogen, Inc., Carlsbad, CA, USA). Single-stranded cDNA was generated from 1 μg of total RNA using TaKaRa PrimeScript™ RT reagent kit. The cDNA products were amplified by real-time RT-PCR using TaKaRa SYBR Premix Ex Taq™ kit and the Bio-Rad IQ5 real-time PCR system, and its analysis software (Applied Biosystems, Carlsbad, CA, USA) was used for data collection and analysis. The primer sequences (Table 1) used for PCR were synthesized by Sangon Biotech Co. Ltd. (Shanghai, China). β-actin was used as the internal control (housekeeping gene).

The PCR protocols were performed as follows: 95 °C for 30 s (initial denaturation), followed by 39–40 cycles at 95 °C for 5 s and 60 °C for 30 s, after which the amplified products were heated from 65 °C to 95 °C at 0.5 °C steps for 5 s. The relative quantification of mRNA expression was analyzed using the 2^−ΔΔCt^ method.

### 4.8. Statistical Analysis

All data are expressed as the means ± standard error (SE) for each group, and the differences between the groups were compared with a two-way ANOVA test using SPSS for Windows, version 19.0 (SPSS Inc., Chicago, IL, USA). The significant differences among the means were determined using Student–Newman–Keuls multiple range tests, and *p* < 0.05 was considered statistically significant.

## Figures and Tables

**Table 1:** The primer sequences used for PCR analysis.

Sequence ID	Name	Sequences
NM_144744.3	*Adiponectin*	Forward: TGGAATGACAGGAGCGGAAG
		Reverse: GCGAATGGGAACATTGGGGA
NM_001276707.1	*SREBP-1c*	Forward: ATCCTGGCCACAGTACCACT
		Reverse: GGAACGGTAGCGCTTCTCA
NM_017332.1	*FAS*	Forward: TCGACTTCAAAGGACCCAGC
		Reverse: ACTGCACAGAGGTGTTAGGC
NM_022193.1	*ACC*	Forward: GTACCGAAGTGGCATCCGTG
		Reverse: TCTCTTCCCGAAGGGCGAAT
NM_013134.2	*HMGCR*	Forward: CCTCCATTGAGATCCGGAGGA
		Reverse: ACAAAGAGGCCATGCATACGG
NM_001145366.1	*PPARγ*	Forward: TGGGGATGTCTCACAATGCC
		Reverse: AGACTCTGGGTTCAGCTGGT
NM_001287577.1	*C/EBPα*	Forward: AGGCCAAGAAGTCGGTGGATA
		Reverse: TCACTGGTCAACTCCAACACC
NM_001007144.1	*ADRP*	Forward: GGCAGGTGACATCTACTCGG
		Reverse: AAAGGGACCTACCAGCCAGT
NM_031144	*β-actin*	Forward: CACCCGCGAGTACAACCTTC
		Reverse: CCCATACCCACCATCACACC
